# Implementation of Comprehensive Genomic Profiling in Ovarian Cancer Patients: A Retrospective Analysis

**DOI:** 10.3390/cancers15010218

**Published:** 2022-12-29

**Authors:** Shira Peleg Hasson, Dov Hershkovitz, Lyri Adar, Miriam Brezis, Eliya Shachar, Rona Aks, Lee Galmor, Yuval Raviv, Shira Ben Neriah, Ofer Merimsky, Edmond Sabo, Ido Wolf, Tamar Safra

**Affiliations:** 1 Oncology Department, Tel Aviv Sourasky Medical Center, Tel Aviv 6423906, Israel; 2 Sackler Faculty of Medicine, Tel Aviv University, Tel Aviv 6997801, Israel; 3 Pathology Department, Tel Aviv Sourasky Medical Center, Tel Aviv 6423906, Israel; 4 Department of Pathology, Carmel Medical Center, Haifa 3436212, Israel

**Keywords:** comprehensive genomic profiling, genomic alterations, matched therapy, ovarian cancer, overall survival

## Abstract

**Simple Summary:**

Ovarian cancer is the third most common gynecologic cancer and the eighth most common cause of death from cancer in women. Comprehensive genomic profiling (CGP) is a test that checks hundreds of genes. Changes in these genes and may help to suggest which anti-cancer treatment may be the most effective for the tested individual. As the test is expensive, and the treatments aimed at treating specific genes related to cancer are both limited and expensive, CGP is not often used. In this study, we investigated whether women with ovarian cancer who had the CGP test had better outcomes (i.e., had longer times with no advancing of disease or lived longer) than women who did not have this test. Our results suggest that women who had the CGP test lived longer than those who did not, but more studies are needed to confirm this.

**Abstract:**

Comprehensive genomic profiling (CGP) allows for the detection of driver alterations at high resolution, but the limited number of approved targeted therapies and their high costs have contributed to its limited clinical utilization. We retrospectively compared data of 946 women with ovarian cancer (11.4% were referred to CGP, and 88.6% served as control) to examine whether CGP provides a prognosis benefit. Patient baseline parameters were similar between the groups. Cox regression analysis adjusted for age, disease stage at diagnosis, and recurrence status showed statistically significantly longer median overall survival (mOS) in the CGP group versus the control (73.4 versus 54.5 months, *p* < 0.001). Fifty-four patients (52.9%) had actionable mutations with potential treatments; twenty-six (48.2%) were treated with matched targeted therapy, showing a trend for longer mOS than the eighty-six women in the CGP group who were not given a suggested treatment (105.5 versus 63.6 months, *p* = 0.066). None of the genomic alterations predicted metastasis location. CCNE1 amplification and KRAS mutations were associated with shorter mOS. Patients with tumor mutation burden ≥4 mutations/megabase had longer mOS. High loss of heterozygosity was associated with longer mOS (99.0 versus 48.2 months, *p* = 0.004). CGP testing may provide both prognostic and predictive insights for treatment of patients with ovarian cancer. Prospective studies of larger cohorts are warranted.

## 1. Introduction

Ovarian cancer is the third most common gynecologic cancer and the eighth most common cause of death from cancer in women around the world [1,2]. The five-year survival rate is 30–45% across the globe [3]. Despite vast investments in the development of genetic and therapeutic agents for ovarian cancer, its treatment remains a great challenge, partly because it comprises a heterogeneous group of malignancies that vary in etiology and molecular biology [4]. The seminal analysis of the Cancer Genome Atlas (TCGA) project, which analyzed 489 high-grade serous ovarian adenocarcinomas, has increased the understanding of ovarian cancer biology, leading to the development of new diagnostic methods and therapeutic approaches. TP53 mutations were found in 96% of tumors and statistically recurrent somatic mutations in nine other genes, including NF1, BRCA1, BRCA2, RB1, and CDK12. Tumors harboring BRCA1/2 and CCNE1 aberrations were found to have an impact on patient survival [5].

The development of next-generation sequencing (NGS)-based diagnostic tools, together with the understanding of the clinical significance of targetable mutations, have enabled the development of personalized treatment approaches. Over two dozen targeted NGS assays are currently available. For example, deleterious BRCA mutations and homologous repair deficiency are predictive biomarkers for the use of poly (ADP-ribose) polymerase (PARP) inhibitors in ovarian cancer [6,7,8,9,10]. In addition, the presence of high microsatellite instability (MSI-high) and mismatch repair can indicate the use of pembrolizumab in uterine cancer, and the presence of programmed cell death protein 1 (PD-1) or a combined positive score can indicate the use of pembrolizumab in cervical cancer [11].

Comprehensive genomic profiling (CGP) [12,13] has been available at our institution since 2011. Patients are usually referred to CGP based on physicians’ clinical judgment and in most cases, after undergoing a blood test for common germline mutations or in the context of screening for a clinical trial.

The relatively high costs of CGP and the limited number of available targeted therapeutics as well as the small number of retrospective studies on CGP have contributed to its relatively low clinical utilization. Nevertheless, evidence supporting the clinical utilization of CGP and the number of targeted agents is increasing for several types of cancers, including hepatocellular carcinoma, melanoma, lung, prostate, and pancreatic cancers [14,15,16,17,18,19].

We conducted a retrospective study to examine whether the use of CGP technology provides a substantial prognostic or predictive benefit for ovarian cancer patients compared to patients who were not referred to this analysis. In addition, we examined if this platform could identify specific biomarkers associated with response to targeted therapy, disease prognosis, or to a specific disease course related to mutations.

## 2. Materials and Methods

### 2.1. Study Protocol and Population

This retrospective observational study was performed at Tel-Aviv Sourasky Medical Center (TASMC). Medical records of 1026 consecutive biopsy-confirmed primary peritoneal cancer or cancers originating in the ovary or fallopian tubes treated at TASMC between 2002 and 2020 were retrospectively analyzed.

The CGP group included records of patients referred to FoundationOne CDx CGP since 2011. The historical control group included patients not referred to any genomic-profiling platforms. This group included patients diagnosed with ovarian cancer at our institution and referred to an analysis of common germline mutations in the BRCA1/2 genes. Patients with wildtype BRCA1/2 genes were subsequently referred to germline BRCA sequencing, and in the last several years—to a somatic BRCA mutation analysis and homologous recombination deficiency testing (myChoice^®^ CDx, Myriad Genetics, Inc. Salt Lake City, UT, USA) on a surgical specimen or biopsy. Notably, some women decided not to undergo this test despite the referral.

Records of patients who were referred to other (non-Foundation CDx) genomic-profiling platforms and records with missing data were excluded from the analysis.

### 2.2. Comprehensive Genomic Profiling

CGP was performed on patient biopsies that were usually obtained during the first debulking surgery and on rare occasions—from biopsies performed later.

FoundationOne CDx (Foundation Medicine, Inc. Beverly, MA, USA) is a CGP platform that applies NGS to in vitro diagnostics with a hybrid capture-based target enrichment approach and whole-genome shotgun library construction to identify all four classes of genomic alterations, including substitutions, insertions and deletions (indels), copy number alterations, and select rearrangements. The typical median depth of coverage is >500×. The CGP panel detects alterations in a total of 324 genes, including all coding exons of 309 cancer-related genes, one promoter region, one noncoding RNA, and select intronic regions of 34 commonly rearranged genes, the coding exons of 21 of which are also included. CGP specimens are also simultaneously profiled for tumor mutation burden (TMB), MSI status, and loss of heterozygosity (LOH) [20].

Sequence data were analyzed using a proprietary software system developed by Foundation Medicine, Inc. Sequence data were mapped to the human genome (hg19) using Burrows–Wheeler Aligner (a program for aligning sequence reads with large-scale reference genomes) [21]. Polymerase chain reaction (PCR) duplicate read removal and sequence metric collection were performed using Picard and SAMtools [22]. Variant calling was performed only in genomic regions targeted by the test.

For the detection of short variants and rearrangements, a de novo assembly was performed. This was done using software to generate a de Bruijn graph, including all k-mers in reads mapping to a particular locus [23]. Each variant had a set of k-mers supporting the variant and a set of k-mers that would support the reference or another variant at the location. Each candidate variant was then scanned against reads in the locus to identify which support the candidate variant, a different variant, or the reference at the location. The supporting reads for each candidate variant were analyzed, and metrics used to evaluate the quality of the variant call were calculated. The final variant calls were made based on a series of quality control filters, which reject a call based on the intrinsic sample noise, the expected noise level for the particular variant, and other known error modes (e.g., sequence homology).

Short variants were reported as a frequency relative to overall depth of coverage; this was denoted as mutation allele frequency (MAF). Alterations were classified as “known”, “likely”, or “unknown” based on their status in the Catalogue Of Somatic Mutations In Cancer (COSMIC). Alterations classified as “other” include truncating events in tumor suppressor genes (splice, frameshift, and nonsense) as well as variants that appear in hot-spot locations but do not have a specific COSMIC association. Variants were classified as variant of unknown significance (VUS) when the significance and impact upon cancer progression were unknown due to a lack of reported evidence and conclusive change in function. It is recognized that some genetic alterations and variants will not impact functionality and do not increase cancer risk.

Genomic rearrangements were identified by analyzing chimeric read pairs. Chimeric read pairs are defined as read pairs for which reads map to separate chromosomes or at a distance of over 2 kilobase pairs (kbp). Pairs were clustered by genomic coordinate of the pairs, and clusters containing at least five chimeric pairs (three for known fusions) were identified as rearrangement candidates. Filtering of candidates was performed by mapping quality (MQ > 30) and distribution of alignment positions (standard deviation > 10). Rearrangements were annotated for predicted function (e.g., creation of fusion gene).

Copy number alterations were detected using a comparative genomic hybridization-like method [24]. First, a log-ratio profile of the sample was obtained by normalizing the sequence coverage obtained at all exons and genome-wide single-nucleotide polymorphisms (SNPs) against a process-matched normal control. This profile was segmented and interpreted using allele frequencies of sequenced SNPs to estimate tumor purity and copy number at each segment.

Tumor content and purity of a sample were derived separately. Board-certified pathologists assessed tumor content through the enumeration of nucleated tumor cells. The assay required greater than 20% nucleated tumor cells to enter into the DNA extraction procedure. This upstream assessment was complemented by the downstream computational calculation of tumor purity just described above. The computational tumor purity assessment was calculated based on SNP allele frequencies and is also used to inform the accuracy of copy number modeling and the calling for several complex biomarkers. The underlying copy number modeling used in making these estimations is a simple two-component system consisting of a mixture of normal diploid cells and aneuploid tumor cells, where the tumor purity corresponds to the fraction of tumor cells in the mixture. The aneuploidy of the tumor cells is modeled as an integral copy number level for each allele of each segment.

To determine MSI status, repetitive loci (minimum of five repeat units of mono-, di-, and trinucleotides) were assessed to determine what repeat lengths are present in the sample. A locus containing a repeat length not present in an internal database generated using >3000 clinical samples, is considered to be unstable. An MSI indicator was generated by calculating the fraction of unstable loci, considering only those loci that achieve adequate coverage for consideration for the sample.

TMB was measured by counting coding short variants present at >5% allele frequency and filtering out potential germline variants according to published databases of known germline polymorphisms, including the Single Nucleotide Polymorphism Database (dbSNP) and Genome Aggregation Database (gnomAD). Additional germline alterations were assessed for potential germline status and filtered out using a somatic-germline/zygosity algorithm [25]. Known and likely driver mutations were filtered out to exclude bias. The resulting mutation number was then divided by the coding region corresponding to the number of total variants counted or approximately 790 kilobases (kb); the resulting number was reported in units of mutations/megabase (mut/Mb). The clinical validity of TMB defined by this panel has been established for TMB-high (TMB-H) as ≥10 mut/Mb as a qualitative status.

For ovarian tumor samples, genomic loss of heterozygosity (gLOH) was measured by the percentage of LOH in the tumor genome. To compute gLOH for each tumor, LOH regions were inferred across the 22 autosomal chromosomes using the genome-wide copy number profile and minor allele frequencies of the germline SNPs. Certain LOH regions were excluded from analysis, including: (1) LOH regions spanning ≥90% of a whole chromosome or chromosome arm, as these LOH events were likely due to non-homologous recombination deficiency mechanisms; and (2) regions in which LOH inference is ambiguous. For each tumor, the percentage of the genome with LOH was computed as 100 times the total length of non-excluded LOH regions divided by the total length of non-excluded regions of the genome. gLOH ≥ 16 was defined as “LOH high”, gLOH < 16 was “LOH low”, and an indeterminable result was defined as “LOH unknown.” In some cases, due to quality control issues, such as low tumor purity, noisy copy number alterations data, and contamination that may affect copy number modeling, it was not possible to accurately calculate LOH. In such cases, LOH was reported as “unknown.”

All CGP reports were presented in a multi-disciplinary team meeting that addressed the opportunities for therapy with approved or investigational new drugs.

### 2.3. Study Measures

Overall survival (OS) was defined as the time between diagnosis and the last follow-up or death. First progression-free survival (PFS) was defined as the period between the end of the first platinum-based chemotherapy administered to the patient and disease progression. Platinum sensitivity was defined as progression more than six months after the end of chemotherapy.

OS was compared between patients with ovarian cancer who underwent CGP and those who were not referred to this diagnostic test. In addition, in the CGP group, OS and PFS were compared between patients who had received a suggested treatment following CGP and those who did not. To evaluate if patients who received CGP-suggested therapies had a clinical benefit, the ratio between PFS on CGP-suggested therapy (PFS2) to the PFS of the previous line of therapy (PFS1) was determined. Clinical benefit was deemed if PFS2/PFS1 was ≥1.3 [26]. To examine whether CGP identifies biomarkers that predict patient outcomes, genomic profiles were analyzed for associations between MSI, TMB, LOH, platinum sensitivity, gene mutations, patient prognosis, and recommended treatments.

### 2.4. Statistical Analysis

All analyses were performed with the IBM SPSS 25.0 software (SPSS Inc., Chicago, IL, USA). Categorical variables were summarized as numbers and percentages and compared using chi-squared test. Continuous variables were summarized as medians and ranges and compared using *t*-test. Median OS and PFS were estimated using Kaplan–Meier survival analyses. The Cox proportional hazards model was used for determining the difference between analysis subgroups with adjustments for age at diagnosis, stage, and recurrence. Statistical significance was set at 5% for all statistical tests.

## 3. Results

### 3.1. Baseline Clinical and Demographic Characteristics of the Study Population and Comparison to the Control Group

Of 1026 consecutive patient records reviewed, 946 were included in the analysis: 108 (11.4%) were referred for CGP (starting in 2011) and the rest (*n* = 838, 88.6%) served as a historical control group in this analysis. All 108 patients referred to CGP were also tested for common germline mutations prior to their referral compared to 64.7% of patients (537/830) in the historical control group. As shown in Table 1, the patients’ baseline characteristics were similar between the CGP and the control groups except for histologic pathology (*p* = 0.002), the frequency of germline vs. somatic BRCA mutations (22.2% germline mutations and 7.4% somatic mutations in the CGP group vs. 35.2% germline mutations and 2.2% somatic mutations in the control group, *p* = 0.001), the proportion of Ashkenazi Jewish patients (69.4% vs. 50.7%, *p* = 0.0007), and the frequency of patients treated with PARP inhibitors as first-line treatment or as part of treating a recurrent disease (30.6% vs. 9.0%, *p* < 0.0001). Over 80% of patients were diagnosed with stage III or IV ovarian cancer.

### 3.2. Comparison of PFS and OS in the CGP and Historical Control Groups

During a median follow-up time of 42.1 months (range, 1.2–308.7) for the CGP group and 39.2 months (0.03–268.8) for the historical control group (*p* = 0.859), median PFS on the first line of therapy was 16.5 months (95% CI 30.4–39.9) and 12.8 months (95% CI, 16.4–27.5), respectively (*p* = 0.082). Statistically, platinum sensitivity was not significantly different between the CGP and historical control group (80.0% vs. 77.6%, *p* = 0.658).

Median OS was also similar for the CGP and historical control groups (69.2 months [95% CI, 63.6–76.0] and 73.4 months [95% CI, 57.9–93.3], respectively, *p* = 0.595). However, Cox regression analysis adjusted for age, disease stage at diagnosis, and recurrence status showed statistically significantly longer median OS in the CGP group compared to the historical control group (73.4 months [95% CI 56.6–93.3] vs. 54.5 months [95% CI 50.6–62.2], *p* < 0.001).

### 3.3. Analysis of the CGP Group

The median time for referral to CGP was 26.5 months (range, 0.4–418.9). Most patients (84/108, 77.8%) were referred to CGP after disease recurrence. Among the 108 patients who had CGP results, the most common gene mutations were TP53 (75.9%), followed by BRCA1 (19.8%), CCNE1 (18.1%), KRAS (11.2%), BRCA2 (10.3%), and MYC (10.3%).

Fifty-four patients (50.0%) had actionable mutations with potential treatments. Twenty-six (48.2%) were treated with matched targeted therapy. Four patients (7.4%) died prior to receiving therapy, and the rest (28/54, 44.4%) could not receive therapy because it had not been approved yet or because they could not afford the recommended therapy.

**Figure 1:**
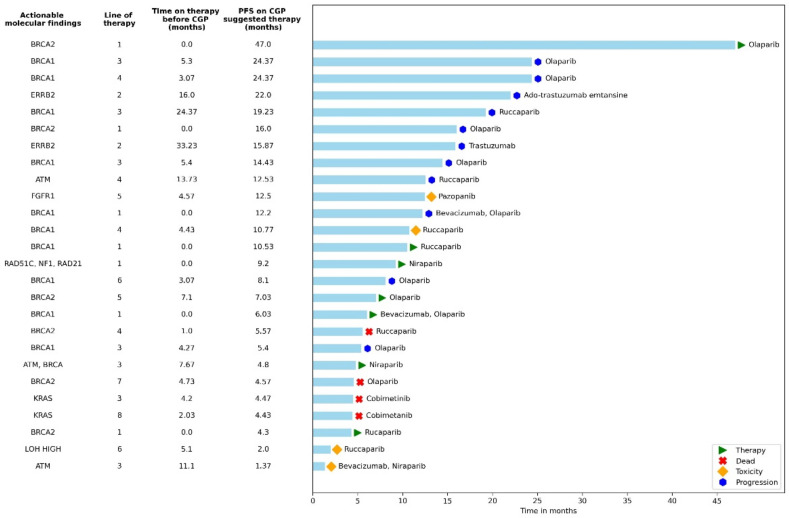
Actionable mutations alongside progression-free survival for patients with molecularly matched therapies given as ≥second-line therapy. The swimmer plot highlights specific regimens documented in patients with actionable genetic alterations. Reasons for discontinuing therapy are shown in the key. This figure provides examples of actual therapies selected in patients with actionable mutations. All 26 patients who received a matched therapy are represented here. PARP inhibitor medications were given to the patients as a maintenance therapy, thus marked accordingly with an asterisk in the line of treatment. Time to disease progression based on matched therapy was longer than the time to disease progression for the therapy prior; however, the results were not significant. CGP = comprehensive genomic profiling, PFS = progression-free survival.

Baseline characteristics (age, histology, disease stage follow-up time) of the 26 patients given CGP-suggested matched therapies were not significantly different from those in the CGP group that did not receive CGP-suggested matched therapy. Figure 1 is a swimmer plot showing PFS in the patients with actionable mutations who received CGP-suggested therapies. The median number of prior therapies in this group was 3 (range 1–8). Most patients treated with suggested therapies had BRCA1 or BRCA 2 mutations (17/26, 65.4%) and were treated with PARP inhibitors (olaparib, niraparib, or rucaparib) as maintenance therapy following first-line therapy or recurrence (21/26, 80.8%); sixteen of these patients were treated with PARP inhibitors following the recommendation of the CGP test. Of note, 20 of the 82 (24%) patients who were not treated with matched targeted therapy also received PARP inhibitors prior to undergoing CGP.

**Figure 2:**
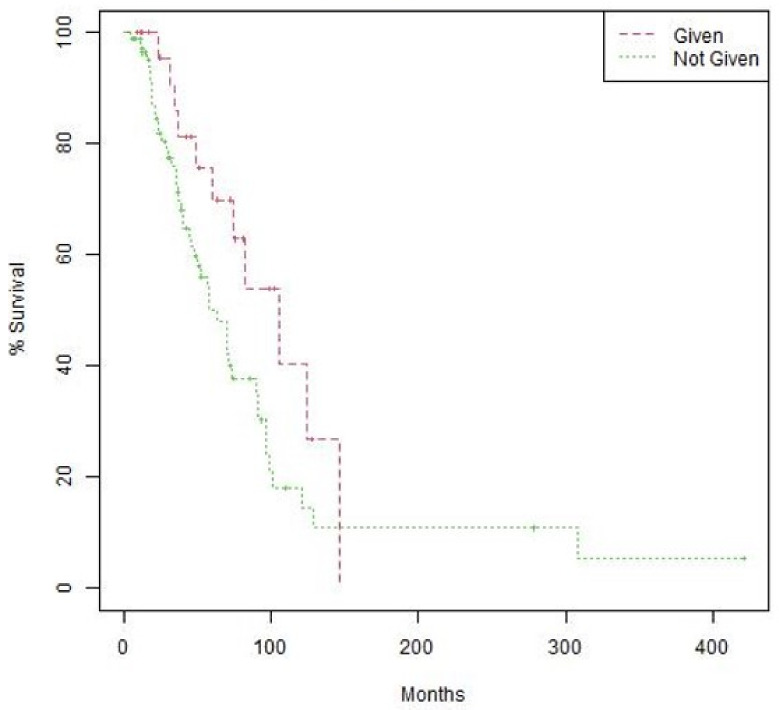
Overall survival (OS) by treatment with CGP-suggested therapy. Kaplan–Meier curves comparing OS of patients treated with therapies suggested by CGP vs. those who were not treated by CGP-suggested therapies. OS = 105.5 months (95% CI 74.4-not reached) vs. 63.6 months (95% CI 48.2–90.9). Log-rank *p* value 0.066.

The women treated with suggested matched therapy had longer median OS with more of a trend for statistical significance than the median OS of the 82 women in the CGP group who did not have an actionable mutation or were not given a CGP-suggested therapy (105.5 months [95% CI 74.4-not reached]) vs. 63.6 months [95% CI 48.2–90.9], *p* = 0.066); Figure 2). Patients on CGP-suggested therapies had longer median PFS when compared to the observed PFS on the immediate prior line but with no statistically significant difference (8.1 months [95% CI 7.5–15.3) vs. 5.2 months [95% CI 4.9–11.6], *p* = 0.290). Of the 22 PFS-evaluable patients who received CGP-suggested therapies, 10 (45.6%) had a PFS2/PFS1 ratio <1.3, which may indicate a benefit to CGP-suggested therapy, and 12 (54.6%) had a PFS2/PFS1 <1.3. These two groups were not distinguishable by age (*p* = 0.096), those who had disease stage IV (0.841), or the time to CGP testing (0.587).

### 3.4. Effect of Biomarkers on Overall Survival

We attempted to find a relationship between the presence of specific biomarkers or mutations and prognosis of patients in the CGP group. No correlations were found between single genomic alterations (GA) and metastasis sites. Multivariate regression analysis adjusted for age at diagnosis and disease stage (III/IV) showed patients with CCNE1 amplifications had statistically significant shorter median OS compared to those without this mutation (45.2 months [95% CI, 35.8-NE) vs. 73.4 months (95% CI, 57.9–100.9], *p* = 0.035). Patients harboring the KRAS mutation also had significantly shorter median OS by multivariate regression adjusted for age at diagnosis and disease stage (III/IV) (40.3 months [95% CI, 17.3-NE] vs. 70.6 months [95% CI, 57.9–97.1], *p* = 0.014). No difference in PFS was found between patients with or without these GA.

We tried to evaluate if specific GA predict platinum sensitivity. Multivariate regression analysis adjusted for age at diagnosis and disease stage (III/IV) showed that patients harboring BRCA1/2 mutations had a trend for a statistically significant higher odds ratio (OR) for platinum sensitivity (OR = 1.17 [95% CI, 0.99–1.39], *p* = 0.062). Patients harboring a KRAS mutation showed a statistically significant lower OR for platinum sensitive disease (OR = 0.72 [95% CI, 0.56–0.92], *p* = 0.010), and patients with CCNE1 amplifications showed a trend for a statistically significant lower OR for platinum sensitive disease (OR = 0.84 [95% CI, 0.68–1.03], *p* = 0.089).

**Figure 3:**
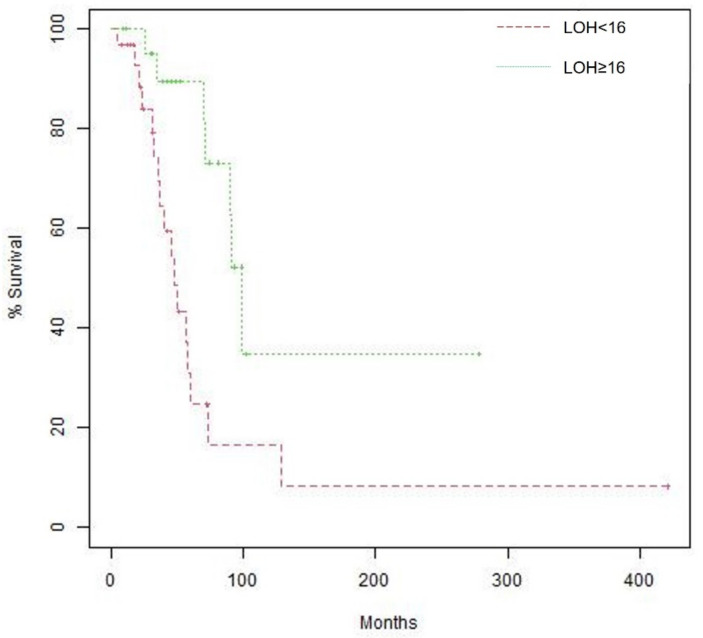
Kaplan–Meier curves of overall survival (OS) according to LOH, Median OS is 48.2 months (95% confidence interval [CI], 36.4 –129.3) for patients with LOH < 16 vs. 99.0 months (95% CI, 90.3—not reached) for patients with LOH ≥ 16. Log-rank *p* value 0.004.

Fifty-three patients had data on LOH: 31 (58.5%) had LOH < 16, and 22 (41.5%) had LOH ≥ 16. A significantly greater proportion of patients harboring BRCA mutations had LOH ≥ 16 compared to those with wildtype BRCA (10/13, 76.9% vs. 12/40, 30.0%, *p* = 0.008). Survival analysis showed that patients with high LOH had longer median OS than those with low LOH (99.0 months [95% CI, 90.3-not reached] vs. 48.2 months [95% CI, 36.4–129.3], *p* = 0.004, Figure 3). This difference remained significant after adjustment for age at diagnosis and disease stage. Furthermore, survival analysis according to LOH and BRCA status showed that median OS was longer in patients with high LOH compared to those with low LOH regardless of BRCA status.

Information on MSI was available for 47.3% of patients—all had low MSI. No correlation with prognosis was found for MSI.

**Figure 4:**
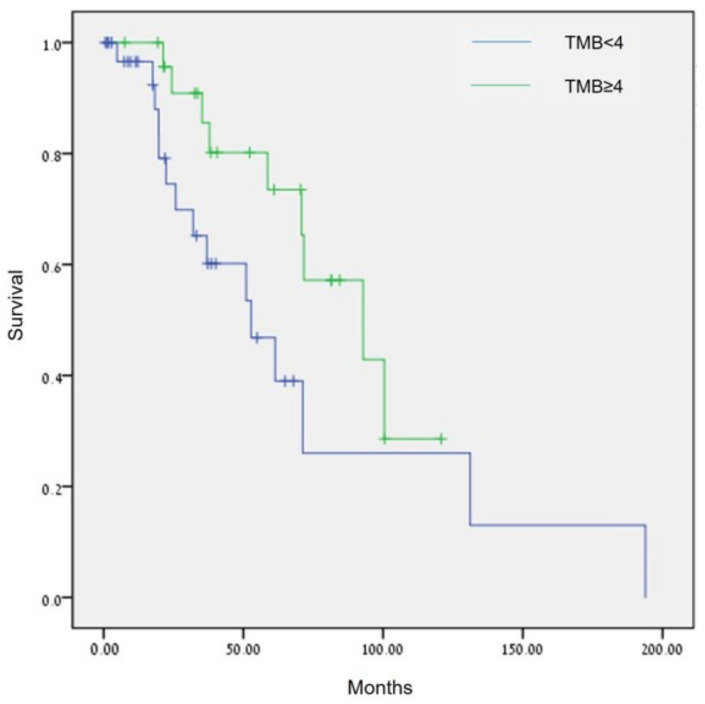
Comparison of OS in patients with ovarian cancer by TMB (<4 mut/Mb vs. ≥4 mut/Mb), *p* = 0.026 (Breslow test).

Sixty-six patients had information on TMB status: 75.8% (50/66) had low TMB status (<5 mut/Mb), and 24.2% (16/66) had intermediate TMB status (5–15 mut/Mb). None had high TMB status. Analysis of TMB using the Breslow test showed patients with TMB ≥ 4 mut/Mb had a statistically significant longer OS compared with patients with TMB < 4 mut/Mb (92.8 months [95%CI, 47.1–138.6] vs. 52.77 months [95% CI, 26.4–79.2], *p* = 0.026, Figure 4).

## 4. Discussion

Comprehensive genomic coverage can support informed molecular-guided treatment decisions as more targeted therapeutics for different biomarkers become available [27,28]. This retrospective real-world study suggests CGP testing might provide both prognostic and predictive insights for patients with ovarian cancer. The frequency of the common GA found in the CGP analysis, namely, TP53, BRCA1, and BRCA2 were similar to those reported by TCGA (Table 2).

CGP testing was associated with improved patient survival compared to patients who were not referred to CGP. Furthermore, patients treated with CGP-suggested therapies had longer median OS than those who received non-personalized treatments, although this is not conclusive. However, the difference in OS between the group of patients treated with matched therapies and those who did not, may have also been affected by the OS of 24% (20/82) of patients who did not receive CGP-matched therapy but received PARP inhibitors prior to undergoing CGP.

Among the molecular biomarkers examined, BRCA, CCNE1, and KRAS, as well as TMB and LOH were predictive for OS. However, due to the small patient sample, a study with a larger patient population is warranted.

In the current analysis we found that physicians usually referred patients to CGP after disease recurrence. As BRCA mutational analysis is currently much cheaper than CGP and performed as standard of care in patients with a known family history of ovarian or breast cancer; all patients in the CGP group had a known germline BRCA status prior to undergoing CGP. Therefore, performing CGP may not have added benefit for those with known germinal BRCA mutations. Several studies, which have evaluated the effects of germline BRCA1/2 mutations on epithelial ovarian cancer prognosis, have shown that both PFS and OS were significantly improved in patients with BRCA mutations [31,32,33]. Analyses of TCGA showed that BRCA2 mutation, but not BRCA1 mutation, was associated with significantly improved OS and PFS [34]. Other studies reported that only OS, but not PFS, was significantly longer in the BRCA mutation group compared to the wild-type BRCA group [35,36,37]. In a study conducted in Korea, BRCA mutations were related to improved PFS but not to OS. In a nationwide study conducted in Israel, improved long-term survival was observed in BRCA1/2 mutation carriers compared to non-carriers [38].

Among the other GA found by CGP profiling, patients with CCNE1 amplification or KRAS mutations had shorter median OS compared to those without these GA. Moreover, multivariate regression analysis adjusted for age at diagnosis and disease stage (III/IV), showed that patients with GA in CCNE1 and KRAS were more likely to have disease resistant to platinum-based therapy, while those harboring BRCA1/2 mutations had statistically significant higher OR for platinum sensitivity.

CCNE1 amplified tumors accounted for 19% of all ovarian cancer samples included in the TCGA PanCan 2018 dataset [39]. CCNE1 amplification has been identified as a primary oncogenic driver in a subset of high-grade serous ovarian cancer. Our findings of shorter OS in patients with CCNE1 are in line with other studies that have found a correlation between CCNE1 amplification in epithelial ovarian cancer and poor outcomes. [40,41]. Furthermore, tumors with CCNE1 amplification are more likely to be resistant to platinum-based cytotoxic agents [42].

Similarly, the presence of a KRAS-variant has also been suggested as a biomarker of poor outcome in epithelial ovarian cancer likely due to platinum resistance. Ratner et al. found the KRAS-variant was a statistically significant predictor for platinum resistance for epithelial ovarian cancer patients of all ages in a multivariate logistic regression analysis controlling for residual disease remaining after cytoreductive surgery, stage, histology, age, and grade [43].

Other potential biomarkers for disease prognosis attained by CGP testing were LOH, TMB, and MSI and may suggest primary therapy modulation. High LOH was associated with longer median OS. The combination of LOH and BRCA mutations was more predictive of OS. Patients with high LOH/BRCA mutation were likely to have the longest median OS. High LOH/BRCA mutation has also been shown to be predictive of better PARP inhibitor treatment efficacy and other therapies [44].

Most patients (75.8%) had a low TMB status. TMB quantifies the mutations as a tumor; it is defined as the total number of replacement and indel mutations per basic group in the exon-coding region of the assessed gene in the tumor cell genome [45]. TMB is usually divided into high and low categories with TMB-high defined as >10 mut/Mb of DNA [46]. It has been suggested tumor types with high TMB correspond to greater generation and presentation of tumor-specific neoantigen, thereby affecting the degree of immune response [47]. At present there are no standards for calculating and reporting TMB and no standardization across platforms or laboratories [48,49]. Furthermore, the threshold for defining high versus low TMB for treatment decisions differs by cancer type and may also differ by immune therapy [49]. Although a TMB-high phenotype has been shown to predict a response to immune checkpoint inhibitors in solid tumors [49], in studies of ovarian cancer, TMB did not predict response to immunotherapy [46]. Moreover, ovarian cancer is considered as having a TMB-low phenotype with a mean TMB of 5.3 mut/Mb and a median TMB of 3.6 mut/Mb and [50,51]. Using the Breslow test, we demonstrated that patients aged ≥ 60 years with a TMB ≥ 4 mut/Mb had a statistically significant better OS compared with those with a TMB <4 mut/Mb (*p* = 0.042). Fan et al. reported that higher TMB was associated with better OS and PFS in patients with ovarian cancer [52]. Bi et al. found a statistical correlation between TMB and FIGO stage, grade, and tumor residual size of 397 patients with ovarian cancer in the TCGA database. They have also observed that a high TMB is associated with better clinical outcomes of ovarian cancer and have suggested that high TMB can induce the activation of antitumor immune cells in ovarian cancer due to higher infiltrating activated memory CD4+ T cells, follicle-assisted T cells, and M1 macrophages, which play an essential role in antitumor activity [45]. This analysis indicates that TMB may be used for evaluating ovarian cancer prognosis.

In the current study, patients with determinable MSI had a low MSI status. The presence of low TMB and MSI may relate to the low effectiveness of immunotherapies for patients with ovarian cancer [53]. Vanderwalde et al. reported a lack of overlap of MSI and high TMB in several cancer types; specifically, MSI-H cases that were not TMB-H or PD-L1-positive occurred in significant percentages of ovarian (24%), neuroendocrine (57%), and cervical (33%) cancers [54]. Therefore, the use of several biomarkers together may improve the appropriate selection of patients for treatment.

To further investigate whether the suggested treatments may contribute to the longer median OS observed in the CGP group, the PFS of patients who were treated with the suggested therapy was longer compared with their previous line of treatment but not statistically significantly different, which can be explained by the small number of patients who received targeted treatments. Another measurement of treatment benefit is a PFS2/PFS1 ratio greater than 1.3 [26]. Among those who had received CGP-suggested therapies, 45.6% had a PFS2/PFS1 > 1.3. In a single-center study that reviewed the clinicopathologic and outcome data of 347 consecutive patients with advanced solid malignancies, a significantly higher percentage of patients who had received CGP-matched therapies had a PFS2/PFS1 > 1.3 compared to those who did not receive matched therapies (45.3% vs. 19.3%) [55]. Therefore, CGP-matched treatments may have prognostic value for patients with ovarian cancer, but this should be evaluated in studies with a larger patient population.

The results of this study should be interpreted with caution since this study was nonrandomized, retrospective, and with a relatively small sample of patients. CGP was not reimbursed; therefore, physicians probably referred mostly patients from higher socioeconomic statuses, who were willing to pay for the test, or those who had private health insurance. In most cases, the CGP suggested therapies were also not covered by the patients’ medical insurance and therefore not reimbursed. The patient population usually had good performance status. Hence, socioeconomic factors, selection bias, cost, homogenous patient population, and availability of medications should be considered when interpreting these results. Thirty-five percent of patients in the historical control group did not have a known BRCA status; most of them did not want to undergo testing. Moreover, until 2004, this test was not reimbursed by the public health plans, and until recently it took over six months to receive the test results from public health plans. Due to the long wait for BRCA test results, sometimes it was easier to obtain CGP results. Due to the small sample size, we could not analyze if there is added benefit to patients with known germline mutations who also had CGP.

## 5. Conclusions

This real-world analysis showed CGP testing provided an OS benefit for patients with ovarian cancer. BRCA and high LOH were both associated with longer response to PARP inhibitors and longer median OS. Of all the GA found, CCNE1 and KRAS were associated with worse prognosis and may suggest resistance to platinum-based therapy. MSI and TMB were mostly low and intermediate and were not associated with outcomes; however, TMB ≥ 4 mut/Mb may be indicative of better prognosis in patients with ovarian cancer. Therefore, CGP testing may provide both prognostic and predictive insights for treatment of ovarian cancer. Prospective studies in larger cohorts are warranted.

## Figures and Tables

**Table 1:** Patient Baseline Characteristics and Demographics.

Parameter	CGP N = 108	Control N = 838	All N = 946	*p* Value
Age at diagnosis (years)	62.7 (34.1–80.2)	61.3 (21.5–93.1)	61.4 (21.5–93.1)	0.373
Stage				
Stages I + II	11/107 (10.3%)	127/824 (15.4%)	138/931 (14.8%)	0.358
Stage III	76/107 (71.0%)	560/824 (68.0%)	636/931 (68.3%)	
Stage IV	20/107 (18.7%)	137/824 (16.6%)	157/931 (16.9%)	
Histology				
Serous papillary	84/107 (78.5%)	529/820 (64.5%)	613/927 (66.1%)	0.002
Endometrioid/poorly differentiated	17/107 (15.9%)	265/820 (32.3%)	282/927 (30.4%)	
Mucinous/clear cell/carcinosarcoma	6/107 (5.6%)	26/820 (3.2%)	32/927 (3.5%)	
*BRCA* mutation status				
*BRCA* wildtype	76 (70.4%)	343/546 (62.8%)	419/654 (64.1%)	0.309
*BRCA*1 mutation	22 (20.4%)	146/546 (26.7%)	168/654 (26.7%)	
*BRCA*2 mutation	10 (9.3%)	57/546 (10.4%)	67/654 (10.2%)	
Unknown	0 (0%)	297/837 (34.7%)	290/945 (30.7%)	
Mutation				
Germline	24 (22.2%)	193/548 (35.2%)	217/656 (33.1%)	0.001
Somatic	8 (7.4%)	12/548 (2.2%)	20/656 (3.1%)	
Ethnicity				
Ashkenazi Jewish	75 (69.4%)	422/832 (50.7%)	497/940 (52.9%)	0.0007
PARP inhibitors	33 (30.6%)	75 (9.0%)	108 (11.4%)	<0.0001

Categorical variables are shown as numbers and percentages and continuous variables are shown as medians (ranges). The denominator is indicated if it was different from the number of the entire patient group.

**Table 2:** Comparison of common mutations found in TCGA * and in the current study.

	Current Study N = 108			PanCancer Atlas TCGA Ovarian * N = 489		
	Mutation	Number of Samples	(%)	Mutation	Number of Samples	(%)
1	TP53	88	(81.5)	TP53	306	(95.9)
2	BRCA1	23	(21.3)	TTN	62	(17.0)
3	CCNE1	21	(19.4)	BRCA 1	37	(11.7)
4	KRAS	13	(12.0)	BRCA2	35	(10.8)
5	BRCA2	12	(11.1)	USH2A	20	(6.3)
6	MYC	12	(11.1)	CSMD3	19	(6.0)
7	NF1	9	(8.3)	FAT3	19	(5.7)
8	PIK3CA	9	(8.3)	MUC16	20	(5.7))
9	RB1	8	(7.4)	LRP2	16	(4.7)
10	ERBB2	6	(5.6)	RYR2	15	(4.4)
11	ARID1A	6	(5.6)	HMCN1	15	(4.4)
12	APC	6	(5.6)	LRP1B	14	(4.0)

* Information was obtained through the cBioPortal for cancer genomics [29,30]

## Data Availability

The data presented in this study are available on request from the corresponding authors. The data are not publicly available due to privacy restrictions.
